# Editorial: Death and Mourning Processes in the Times of the Coronavirus Pandemic (COVID-19)

**DOI:** 10.3389/fpsyt.2022.922994

**Published:** 2022-05-26

**Authors:** Lydia Giménez-Llort, Virginia Torres-Lista, Efosa K. Oghagbon, Heloisa Vicaino Fernandes Souza Pereira, Maria-José H. E. Gijsberts, Sara Invitto

**Affiliations:** ^1^Department of Psychiatry and Forensic Medicine, School of Medicine, Universitat Autònoma de Barcelona, Barcelona, Spain; ^2^Institut de Neurociències, Universitat Autònoma de Barcelona, Barcelona, Spain; ^3^Dirección de Investigación, Catholic University Santa Maria La Antigua, Panama, Panama; ^4^Scientific Research Center for Social Sciences (CENICS), Panama, Panama; ^5^College of Health Sciences, Benue State University, Makurdi, Nigeria; ^6^Departament of Pediatrics, Rio de Janeiro State University, Rio de Janeiro, Brazil; ^7^End of Life Research Group, Vrije Universiteit Brussel and Universiteit Gent, Brussels, Belgium; ^8^INSPIRE LAB - Laboratory of Cognitive and Psychophysiological Olfactory Processes, Department of Biological and Environmental Sciences and Technologies, University of Salento, Lecce, Italy

**Keywords:** coronavirus, death and dying, grief/loss, mourning (bereavement), secondary impact, spirituality, resilience, end-of-life

Two years after the COVID-19 outbreak (WHO), and already in the 6th wave in some countries, we feel overwhelmed by the portrait of “our world in data” (Figure 1, top). The devastating total numbers (504 M cases and 6.2 M deaths) can hardly attempt to quantify the human dimension of a world map full of spots that feel like black holes. As of today, April 17, 2022, the most recent scenario unveils the pervasive emptiness, with 44.888 new persons worldwide that confronted death in the last 14 days and the start of a bereavement period for their beloved families to grieve and mourn their loss. Yet, the non-COVID deaths associated with the secondary impact of this pandemic will be hard to record. Nevertheless, the number of recovered patients reported worldwide and the extraordinary scientific efforts to draw a vaccine map, are new seeds of hope in this COVID-19 outbreak, one of the deadliest pandemics in human history.

**Figure 1:**
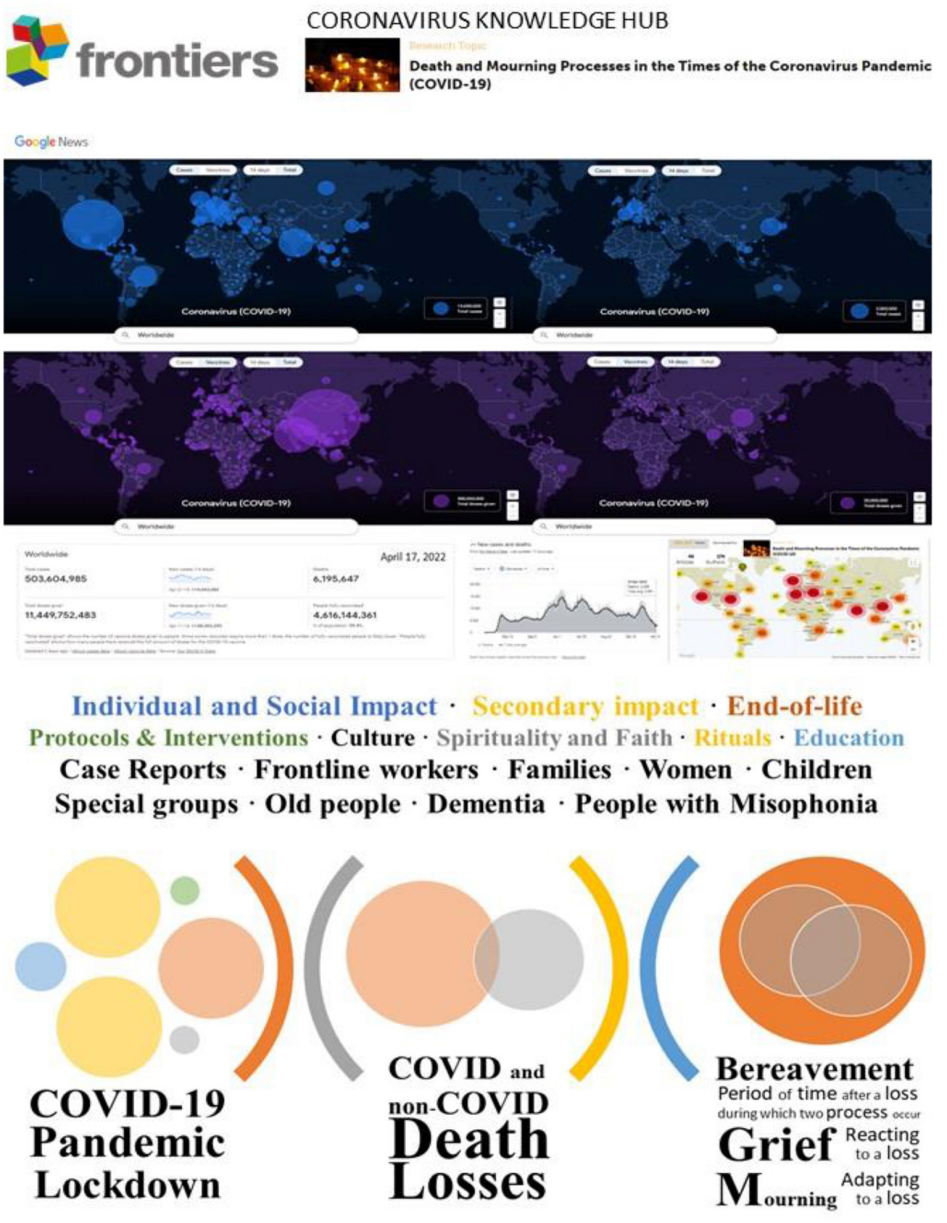
Frontiers Research Topic on “Death and mourning processes in the times of the Coronavirus pandemic (COVID-19)”: “Our world in data” Coronavirus World Map, Impact and Thematic Contents. **(Top)** World maps from Google News – Coronavirus (COVID-19) (8) (source: https://news.google.com/covid19/map, http://oneworldindata.org, accessed April 17, 2022) for total (left) and last 14 days (right) cases of COVID-19 (blue map on the top) and vaccines (purple map on the middle) from the start of COVID-19 outbreak until today, April 17, 2022. **(Middle)** From left to right: Worldwide records for Total cases, New cases (14 days, Apr 2–5), and Deaths; Total doses given, New doses given (14 days, Apr 1-14), and People fully vaccinated in total amount and percentage of the population. “Total doses given” shows the number of vaccine doses given to people. Since some vaccines require more than 1 dose, the number of fully vaccinated people is likely lower. “People fully vaccinated” shows how many people have received the full amount of doses for the COVID-19 vaccine. Updated 2 days ago. Source: Our World in Data. **(Middle)** New cases and death from the beginning of the pandemic until Apr 15, 2022. Right panel: Impact of the Research Topic “Death and Mourning Processes in the Times of the Coronavirus Pandemic (COVID-19)” per number of articles, authors, and demographics (map of views) of the impact (source: https://www.frontiersin.org/research-topics/14142/death-and-mourning-processes-in-the-times-of-the-coronavirus-pandemic-covid-19#impact, accessed April 17, 2022) (9). **(Bottom)** Thematic contents and scenarios of Research Topic “Death and Mourning Processes in the Times of the Coronavirus Pandemic (COVID-19).”

Looking back to those dreadful early months in China, Italy, and Spain that warned what would happen worldwide, this Research Topic emerged as the scientific readout of a tend-and-befriend copying (1) that gathered together online an international group of 12 people handling and analyzing the unprecedented and devastating pandemic scenario through the lens of psychiatry, neuroscience, geriatric, palliative care, psychology, medicine, nursery, education, sociology, pediatric and pharmacy. At that time, reports from official sources and trustful academic and social media were the only information available and primarily focused on the virus, while aspects related to its actual impact could hardly be heard (2–4). Thus, from our respective countries (Argentina, Belgium, Brazil, India, Iran, Italy, Netherlands, Nigeria, Panama, and the USA), we realized the taboo of death at the individual and social levels would worsen the impact on the public health emergency under the pressure of chronosystem (5) and the minatory shadows of a secondary impact and multiple mourning that could already be foreseen (6, 7). The goal of this Research Topic was to face this taboo with an international call to address death and mourning processes from different multidisciplinary perspectives and cultures. We encouraged immediate opinions, perspectives, and reviews that would open discussion about the challenges and solutions before original research reports could arrive. This Research Topic compilates the efforts of 174 authors from 22 countries (Argentina, Australia, Austria, Brazil, China, Iran, Ireland, Israel, Italy, Japan, Nigeria, Norway, Pakistan, Panama, Portugal, Romania, South Arabia, Spain, Sweden, Switzerland, UK, and the USA) that joined us with 46 scientific papers in this goal. Their work was reviewed by 89 peers that added 14 other countries to the international scientific discussion panels (Bangladesh, Canada, France, Germany, Greece, Indonesia, Lebanon, Poland, Shri Lanka, Singapore, South Africa, South Korea, Taiwan, and Tunisia). This effort, from five continents, deserved the attentive look of 280,507 views and 36,501 downloads worldwide and a 674 total altimetric (Figure 1, middle). The editorial follows the structure of the different proposed thematic aspects (Figure 1, bottom), and we have cited the first authors with names and surnames and their countries to emphasize their personal commitment from different parts of the world, to provide not only technical but also spiritual and educational tools to mitigate the present and future impact of COVID-19 in our multiple mourning process as individuals and/or as a society.

## Cross-Cultural Bio-Psycho-Social Perspectives

The universality of death and mourning processes is shaped by cultural perspectives (10), whose relevance is enhanced in devastating scenarios. In the current pandemic, these processes mirror the singularity of individual and societal impacts that can be alleviated through resistance and resilience. These two natural human capacities are related to demographics, available resources, and life stress (11). Person-centered variables and socio-contextual factors such as previous experiences, beliefs, education, and culture can promote them. Therefore, cross-cultural bio-psycho-social perspectives confronting multiple losses, death, and mourning can make dying, as Bermejo (Spain) said in his Opinion, “an archipelago, which is characterized precisely of being united by what it separates.”

Bilimoria (Australia), in his “Hindu response to dying and death,” argued and pleaded for a paradigm shift in the discursive and practical approaches to the COVID-19 pandemic, looking through the broad lens of Hinduism. That may help therapists and care workers develop a deeper understanding of cultural and religious beliefs. Also, to improve clinical practice and therapy sessions by expanding their toolkit with interventions (yoga, mindfulness, etc.) already proven to positively affect other severe health and life-threatening conditions. The review “Understanding grief during the first wave of COVID-19 in the United Kingdom” by Fang and Comery (UK) examined the first available reports from trusted media and carefully selected academic literature. They adopted a hypothetical approach to explore the experiences of death and grief in the earliest period of the pandemic. The diverse and dynamic nature of the grief needs and responses at an individual and societal level was analyzed comprehensively. They revisited the ideas of “good” and “bad” deaths and bereavement support, thus providing foundational understandings for the new challenges and opportunities, also relevant for other socio-cultural contexts. In the Perspective article “Managing Grief of Bereaved Families During the COVID-19 Pandemic in Japan,” Matsuda et al. (Japan) described different cultural aspects linked to the Japanese situation and how they managed psychological distress due to the distance from a sick or dead family member. The authors suggested training methods for telepsychological interventions that could allow a face to face treatment. From an autoethnographic and ethnographic analysis, Ali (Austria) discussed the challenge that the shift of meaning of a “normal” dead body to a “viral” dead body represents for cultures, people, and governments. He described significant differences between customary death rituals and ceremonies of a dead body during ordinary situations in Pakistan and those performed during current times by government mandate and warned about the harmful impact on mental health. In the “Global Financial Crisis, Smart Lockdown Strategies, and the COVID-19 Spillover Impacts: A Global Perspective Implications From Southeast Asia,” Wang et al. (China) provided a detailed analysis of the critical factors of the global health emergency and the secondary impact on mental wellbeing and socio-economy, with global perspective implications by examining Africa, America Asia, Australia, Europe, and the Middle East, and presenting Pakistan as a case study. Non-pharmaceutical interventions were proposed to formulate smart lockdown strategies to restart economic activities and help progress toward the next normal in society.

## The Role of Spirituality, Faith and Meaning of Life

This was the thematic content receiving the first Opinions that emerged as a claim, as highlighted in the subsequent contributions. The first paper arrived from a Spanish center for the humanization of health to translate the reflections on dignified and humanized death offered in their books into the current landscapes. In a rosary of spiritual thoughts and faith, Bermejo revealed the transcendental relevance of accompaniment in grief in times of coronavirus. Similarly, the opinion by Junior et al. (Brazil) poetically described real scenarios hidden in the news: Silent cries intensify the pain of the life that is ending in a scenario where COVID-19 was literality robbing families of the chance to say a final goodbye. In a second opinion, Júnior et al. summarized what the literature tells about the use of spirituality as a tool to face the negative symptoms of various pathologies. Its influence on mental health results from several factors, it improves therapeutic adherence, self-esteem, and self-control and strengthens family/friendship ties and intellectuals. Using other tools such as beliefs allows us to reduce the adverse effects of the pandemic, social isolation, and routine deprivation, which is necessary to establish a sense of security and normality. Religiosity as a resilience factor in stressful conditions was also addressed by Testoni et al. (Italy, Israel, USA). They investigated the mourning and management of the health emergency in a priestly community in the most affected Italian region. They depicted their psychological experiences related to the contagion and the eventual death of colleagues and the resilience strategies activated during the process. The thematic analysis unveiled how religious faith transformed fear and pain into motivation to strengthen the social bond, guide the community toward the goals of spirituality, and the ways of prayer and reflection about the finiteness of life. In a second work, Testoni et al. (Italy, Israel) studied the evolution of the experience of elective abortion in nine non-practicing Catholic women. In a third assessment, coinciding with the worst period of Italy's COVID-19 crisis and the Easter declaration against abortion, the conflicts between women's religiosity and their sense of free will, aggravated their physical, relational, and psychological suffering, similar to perinatal grief.

Borghi and Menichetti (Italy) also developed their research study in Lombardia, where the higher percentual of deaths due to COVID-19 occurred in 2020. They described spontaneous strategies that family members adopted to cope with a relative's loss and manage stressful conditions in such severe circumstances. Also, Coppola et al. (Italy) studied “Spiritual Wellbeing and Mental Health During the COVID-19 Pandemic in Italy” to understand the role of spirituality and religiosity in reacting to the pandemic situation, and in particular on the physical and mental health of the people involved. Spirituality and religious practices were protective factors connected with psychological, mental and physical health. Finally, Bartholo and Proença (Brazil), in their brief essay entitled “Sister Death, Cousin Grief: Modes of Presence Before Life and Death” shared a mode of thinking in contrast to contemporary happy modernity that stands for expectations of limitless prosperity and progress. They argued for philo-zoe (life) rather than philo-sophy. They disclosed about what to be born (“brother birth”) and to die (“sister death”) is, the enjoyment of incoming alterity (“cousin expectation”) or the apologetical rite for alterities passed away (“cousin grief”).

## Risk Groups, Challenges, and Controversies in Regulations and Priorities Implemented by Governments and Policymakers in COVID-19

The maximum exponential of these thematic aspects could be found in the research work “Under-reporting of COVID-19 cases among indigenous peoples in Brazil: A new expression of old inequalities” by Fellows et al. According to COIAB's reports, the epidemiological analysis of COVID-19 in the Brazilian Amazon home of half a million Indigenous Peoples representing 170 ethnic groups drew a dramatic scenario that deserved international coverage in the news and was part of the documents used to present legal actions against the federal government. This large study desperately urged when Brazil recorded its deadliest month as the health crisis deepened. It provided the first scientific evidence of the Indigenous Peoples' higher vulnerability (136 and 110% higher incidence and mortality, respectively, than the national average) and the abysmal dimension of under-reporting of deaths (103%) by the Brazil Ministry of Health. The geospatial analysis of external threats contributing to the spread of the disease determined the connection between illegal economic activities (deforestation, land grabbing, and mining) as those increasing the risk to get infected or dying from COVID-19 in the Indigenous lands. Public health policies considering the Indigenous movements and associations, and their rights were proposed as the two solutions to guarantee the survival of Indigenous Peoples in the COVID-19 pandemic. A second critical analysis by Ali entitled “Rituals of Containment: Many Pandemics, Body Politics, and Social Dramas During COVID-19 in Pakistan” further explored how the multidimensional faces of pandemic scenarios were dealt. The author developed the “rituals of containment,” a new concept that critically analyses the interplays among religion, science, and politics, to conclude that using religion and science to justify political responses to the pandemic and exert hegemonic power reveals manipulative, discriminatory, and contradictory government policies and practices. On the other hand, in the absence of effective vaccines, Zhou et al. (China, Romania, Saudi Arabia) recommended non-pharmaceutical interventions to respond to the COVID-19 challenges and socio-economic crisis. The combination of two model strategies, such as the suppression strategy (lockdown and restrictions) and the mitigation model, was found to be helpful to decrease the burden and the impact on exhausted health systems.

The challenges and controversies at the beginning of this pandemic also included mask use and the impact of strict confinement (see below). The use of masks in preventing the spread of the infection was controversial since they were considered useless or mandatory depending on the country. Using three different types of software for computer simulation, Johansson (Sweden) presented a software solution to visualize infection spread, identified the most effective timepoints and situations for mask use, and estimated the effect of masking the general population considering the protective effects of different types of masks, their side-effects and ways to improve masks and masking. His simulation analysis demonstrated that masking the general population could limit the spread of SARS-CoV-2 in a country and allowed to identify opportunities when mask use was cost-effective and safe.

## The Secondary Impact of COVID-19

One of the most singular aspects of this Research Topic was that it offered extraordinary insights into the pandemic, either as personal reflections or singular case reports. The first case report by Arumi et al. provided a first-person experience of delirium of a Spanish physician who, 3 days after being on strict lock-down, acquired COVID-19 and developed bilateral pneumonia with a complicated course of illness. He had delirium with a recall of experiences of reality and unreality, complete disorientation, lack of control, strong emotions, and intense fear of dying, which was significantly distressing. Given the significant prevalence of delirium in COVID-19 patients, the authors anticipated that the delirium burden would be expected on these patients and their families and warned clinicians to evaluate the neuropsychiatric consequences of this condition.

The unprecedented and extreme situations in the current COVID-19 pandemic are risk factors for psychosocial stress for the entire population and have a stronger impact on psychological profiles at risk or those already with mental disorders. Italy and Spain suffered a high mortality rate in Europe that forced a strict lockdown in March 2020 (12). Both factors induced worrisome scenarios addressed by several original research works to provide scientific data to understand, assess their impact and provide clues for better management. Murphy and Moret-Tatay (Ireland, Spain, Italy) studied participants from these two countries who suffered the loss of someone close. They examined the role of country of origin and residence, personality traits, and attitudes in confronting death awareness. Their results showed stability of personality and no differences during grief across countries. Two different clusters were identified for attitudes, despite not differing in personality. Cluster group, neuroticism, age, and sense of belonging to the country predicted fear of contagious diseases, warning about particular psychological needs during grief in persons with higher scores in neuroticism. In a second part of this study, Pérez-Mengual et al. (Spain) further explored the relationship of fear of death between neuroticism and anxiety in a Spanish sample. Biological (sex) and psychological (fear of personal death, neuroticism, and extraversion) variables predicted anxiety. Women displayed higher scores on anxiety and fear of personal death, whereas in men, the fear of personal death mediated the relationship between neuroticism and anxiety. Major stress-related symptoms during the lockdown in Italy were investigated by Invitto et al. of the Italian Society of Psychophysiology and Cognitive Neuroscience. They described the Italian situation during the lockdown and highlighted how self-perception involved great sensorial cross-modality. Disosmia was related to the belief of having had the COVID-19. Moreover, one of the most stress-related symptoms was sleep alteration. Gender differences were highlighted in managing stress, somatization, and pain perception. In fact, women seem to be more affected by the psychophysiological effects associated with the pandemic, presenting a lower resilience. In a translational neuroscience approach using old 3xTg-AD mice mimicking advanced stages of Alzheimer's disease and the devastating scenarios in nursery homes, Muntsant and Giménez-Llort (Spain) demonstrated the impact of social isolation on behavioral and functional profiles. Asymmetric atrophy of the hippocampus, recently described in people with dementia and modeled in their work for the first time, was found to increase with isolation, warning of the severe impact and the urgent need to reconsider and redesign all living conditions, care/rehabilitation interventions, and management of loneliness forced by social distance measures.

The forced strict confinement to hamper the COVID-19 pandemic in Spain seriously affected people suffering from misophonia, a complex neurophysiological and behavioral disorder characterized by intense physiological and emotional responses produced by intolerance to auditory stimuli. Two longitudinal studies by Ferrer-Torres and Giménez-Llort (Spain) provided evidence of the complex burden and gender perspective of the secondary impact of the pandemic on the mental health of this clinical population. The first study recorded an exponential increase in clinical consultations for health, fears, conflicts with neighbors, and learning difficulties. An alarming increase of severity on 8 out of 9 psychopathological items (mainly sleep disorders, hostility, depression, and somatization) was worse in women than men, demanding the development of coping strategies addressing modifiable risk and protective factors and deserving familial/social comprehension and stronger clinical support. In their second study (Ferrer-Torres and Giménez-Llort), a semi-structured interview confirmed the impact of 3 months of strict confinement on enhanced hyper-sensorial sensitivity and fear of getting infected with or dying from COVID-19, general emotional maladjustment, distress, and transitory crisis. Long-term neurophysiological biomonitoring identified a significant increase in physiological arousal and the loss of cardiac coherence in response to triggering auditory stimuli, but also under basal rest/relaxation conditions or even imagined triggering sounds that worsen with the severity of misophonia and in women.

David et al. in a perspective from a team of researchers from different universities in Nigeria, the UK, and the USA gathered by our collaborator Ismaeel Yunusa, depicted the secondary impact of measures to hamper the pandemic and the increase in the number of non-COVID-19 deaths triggered by factors such as domestic violence, suicide, hunger, and the exhausted healthcare system. They analyzed the causes and scenarios to propose a series of solutions through NGOs, governments, and individuals to mitigate these challenges.

The secondary impact of the pandemic with worsening of previous mental health problems, including depression and anxiety, severe socio-economic and personal constraints, and fear of infection, has also provoked cases of COVID-19 related suicides worldwide. A lexical and content analysis by Júnior et al. (Brazil) described how the COVID-19 pandemic impacted suicide behavior and was strongly linked to social isolation and decreased health resources (professionals, opening hours, demand for medications). Furthermore, behind this dramatic situation is that each suicide may affect between 5 nuclear family members and 80 relatives/friends known as “suicide survivors.” They are one of the largest at-risk communities for mental health disorders, including complicated grief, depressive and anxiety disorders, substance-abuse disorders, and suicidal behavior. The review article by Pinto et al. (Portugal) on COVID-19-related suicides was devoted to visualizing this hidden grieving population and showing that feelings of guilt, shame, and social stigma were significant obstacles to reaching for help. They highlighted modifiable risk factors and proposed early identification and intervention, the use of technology, promotion of help-seeking behaviors, and social education on suicide and its impact to tackle stigma.

The opinion “COVID-19 and Disenfranchised Grief” by Alburquerque et al. (Portugal) offered a preliminary view about COVID-19-related deaths being lonely and dehumanized processes for patients and families, warning about the significant risk for disenfranchisement in grief and added burden during bereavement. To promote healthy COVID-19-related grieving, they provided a list of recommendations regarding disenfranchisement imposed externally and self-disenfranchisement. Testoni et al. (Italy, Israel) also provided evidence that grief caused by COVID-19 can be considered both traumatic and de-legitimized and, in this sense, comparable to the effects of ambiguous loss. They are related to the experiences of dehumanization, the lack of contact with the dying relative and the corpse of the deceased, and the absence of funeral rituals.

## Educational Needs on Death and Mourning Process

In contemporary western societies, death is not only a social but also an educational taboo, two shortcomings that were highlighted by this pandemic. Two original research works from academia carried out during the first year of the pandemic illustrated the relevance of death education. The first one, by Testoni et al. (Italy, Israel, Austria), analyzed the psychology students' perceptions of the lock-down experience in a death education course. Three main topics were identified: the removal of death in contemporary culture; the importance of community, ritual, and funeral, and spirituality; and the relevance of death education for future health professionals. Removing these issues exposes people to the risk of being unable to deal with harrowing events, such as those associated with death. The second work, by Giménez-Llort (Spain), was the first original ethnography study of a viral YouTube educational video. “Vuela Mariposa, Vuela,” a children's story for preschoolers about the cycle of life and disenfranchised grief, went viral in Ecuador when the devastating scenarios in its streets were yet unknown. The analyses identified a switch in the users' profiles, engagement, and feedback, from adult Mexican and Spanish women's grief narrative expressions to a broad age Ecuatorian population, including men. The video was part of an academic assignment or used to express critical thinking on life's meaning and societal mourning. This ethnography pointed at YouTube as an educational resource highly sensitive to critical events and fast-spreading among diversified users. Thus, good practices make it eligible as “palliative social media.”

## The Impact of Death From COVID 19 and The Mourning Process on Front-Line Professionals and Their Families

Like soldiers, front-line healthcare workers and researchers have confronted the psychological and physical impact of the COVID-19 pandemic, and many have lost their lives in the battle to fight the pandemic. The perspective by Das et al. (India) outlined the series of stressors and the impact on their mental well-being (depression, anxiety, insomnia, and post-traumatic stress symptoms) but a scarcity of studies on the death and mourning process or complicated grief in front-line workers and their families. They called for investigations to understand these issues and test the effectiveness of the recommendations done to provide psychological support to bereaved families. From Panama, Oviedo et al. presented critical reflections on the role of clinical researchers during COVID-19. They discussed their challenging role in managing emergencies and balancing the individual, scientific and social benefits of research. They suggested how research platforms could be dedicated to generating knowledge, using open data, and aiding in managing mental health issues.

## Challenges and Management of Loneliness, Palliative Care, and Dying Alone

The devastating scenarios of this pandemic have been extreme in palliative care clinical and home settings (13). A critical contribution of this Research Topic is that many papers provided a critical analysis of the impact of restrictions on visiting hospitals and standing by the sick, and new protocols were shared to help others struggling with similar problems. Most importantly, they focused on end-of-life scenarios where the absence of standard rituals on the death/dying process (14) made COVID-19 rob families of the chance to say a final goodbye, increasing psychological suffering and worsening the grieving process as described by Júnior et al. (Brazil). The experience of a palliative care team in Portugal presented by Carvalheiro et al. also exemplified the severe impact of protocols for the general population, the need for preferential circuits for end-of-life patients and their families, and the reinvention of grieving support programs. They warned about inadequate flowcharts for asymptomatic terminal patients and those with “suspected” infection that were submitted to the same restrictions, compromising the safety or resulting in families keeping their terminally ill loved ones at home as long as possible. These questions also affected those admitted to the hospital for other reasons than COVID-19. From Vall d'Hebron University Hospital in Barcelona,  Beneria et al. (Spain) presented the structure, circuit, and functions of their “End of Life (EOL) Intervention Program During COVID-19,” which aimed to dignify EOL and mitigate future complications in grief and prolonged grief disorder. A multidisciplinary team of health social workers and clinical psychologists collaborated with medical teams and nursing staff to implement the program based on family perceptions of EOL care and the restrictions. It was activated in most EOL situations so that relatives could come and say goodbye to their beloved ones, but also had an emotional impact on the EOL team. Júnior et al. (Brazil) also provided insights into the dramatic challenges in palliative and EOL care during the pandemic. They presented a detailed proposal of their adapted SPIKES protocol to ensure effective and honest communication, particularly important in communicating bad news to patients and their families.

## Companion Animals, Mobile and Internet-Based Interventions, and Psychosocial Tools to Mitigate Fear and Grief

“The value of companion dogs as a source of social support for their owners” by Bowen et al. (UK, Spain) was demonstrated in a convenience sample obtained during the COVID-19 stick lockdown in Spain. The study showed that owners shared more activities with their dogs, hugged them more often, and turned to them more as a source of company and comfort. The authors concluded that when a dog is present in a home, it must be an essential resource for social, clinical, and decision-making support in public health.

Grief follow-up is often provided by nurses, volunteers, and social workers, and the type of support can range from handing out a condolence brochure or letter to individual or group support. Borghi et al. (Italy, Norway) presented “A Phone-Based Early Psychological Intervention for Supporting Bereaved Families in the Time of COVID-19” and concluded that teletherapy using mobile/telephone devices facilitated the follow-up of the mourning and can be conceived as an interface between bereavement follow-ups and interventions in a psychological emergency. In their Opinion article, Berthoud et al. (Switzerland) addressed adults struggling with prolonged grief symptoms after an interpersonal loss, by bereavement or separation/divorce, both increased in this pandemic. They presented LIVIA 2.0, a new online program for grief based on German and French versions of LIVIA, serving as an intermediary to build new relationships that may help overcome mourning and the treatment gap (not receiving treatment despite mental wellbeing problems). The possible relationships between ambiguous loss, the use of the internet, and prolonged grief risk were discussed by Testoni et al. (Italy, Israel), and the exploration of media portrayals of public sentiment on funerals (Saraff et al.) allowed to recommend health professionals must have intervention protocols, whereas the media must be more empathic on this subject because they reflect on society. In a third qualitative study on emotional catharsis, Testoni et al. suggest cinema as a powerful tool to help people face the intense uncertainty of the pandemic by obtaining a greater understanding of the situation and projecting their fears and uncertainties.

## Special Considerations and Solutions With Children and Adolescents Who are Going Through a Duel During Confinement or Confront a Multiple Mourning

Complicated grief in adolescents is widely underrecognized, often misdiagnosed, and has severe consequences since it has been related to 25% of suicides or 41% of juvenile offenders. In the perspective “It's Complicated—Adolescent Grief in the Time of COVID-19” Weinstock et al. (UK) explain such complexity as unique due to bio-psycho-social factors (increased risk-taking, identity-formation, and limited emotional regulation). They recommended that mental health professionals and organizations respond to the “bereavement pandemic” by fostering collective structures that can provide bereaved youth with a sense of belonging.

Chachar et al. (Pakistan, USA) applied Bronfenbrenner's bioecological models to explore how mutual interaction between a child and various ecological systems determines death perception/cognition and how the pandemic influenced death-related attitudes and understanding during childhood development leading to a life-long impact. “In the Same Storm, but Not on the Same Boat”: by Alburquerque and Santos (Portugal) also warned about children's grief during the pandemic. Recognizing differences between child/adult is essential to adequately attend to the child's needs in a pandemic scenario that has altered the perception of security and predictability essential when looking at the world and relationships. Enhanced empathy and connectedness through this shared experience are possible only if the culture of silence surrounding death is not perpetuated. A case report presented by Santos et al. (Portugal) further contributed to understanding the complex scenarios of children mourning a parental loss during the COVID-19 pandemic and under confinement limitations. They described the therapeutical strategies used through telephone calls and face-to-face appointments and their limitations to finally advocate that policies and rules regarding the pandemic should consider measures to protect mental health, facilitating the grief process.

## Final Considerations

Collected through an extensive Research Topic, these international collaborations described the facets of the different ways of seeing and dealing with the death and mourning processes during the pandemic. They highlighted the great cultural, clinical, and social differences but also a common condition, which is human nature. When writing this editorial, the pandemic is still waiving, but above all, it seems extremely modified compared to its initial condition. The COVID-19 disease has changed due to the different virus variants and the social situation after vaccines were developed and administered. The population no longer faces the stressful effect of an unprecedented pandemic, and individuals and society are more prepared to face, through coping strategies, the event that has now evolved both from a clinical and social point of view. Beliefs regarding COVID-19 have changed and will certainly be investigated in future research. Furthermore, for the first time in the field of scientific dissemination, open access journals have allowed a gold model also leveling the gap present in this type of publication, compared to laboratories with a lot of funding and laboratories with little funding, a gap highlighted just recently (15). This has allowed raising our voices as one to express and analyze death and mourning processes from the five continents. Even science, in an emergency pandemic situation, has been able to face the possibility of doing research without restrictions with highly resilient strategies.

## Author Contributions

LG-L: original idea and writing—original draft preparation. LG-L, SI, VT-L, EO, M-JG, and HP: editorial work. LG-L, SI, and VT-L: writing—review of records and editing. All authors: conceptualization. All authors contributed to the article and approved the submitted version.

## Conflict of Interest

The authors declare that the research was conducted in the absence of any commercial or financial relationships that could be construed as a potential conflict of interest.

## Publisher's Note

All claims expressed in this article are solely those of the authors and do not necessarily represent those of their affiliated organizations, or those of the publisher, the editors and the reviewers. Any product that may be evaluated in this article, or claim that may be made by its manufacturer, is not guaranteed or endorsed by the publisher.
